# MicroRNA-203a inhibits breast cancer progression through the PI3K/Akt and Wnt pathways

**DOI:** 10.1038/s41598-024-52940-5

**Published:** 2024-02-27

**Authors:** Maryam Entezari, Bahram M. Soltani, Majid Sadeghizadeh

**Affiliations:** Department of Molecular Genetics, Faculty of Biological Sciences, Tarbiat Modares University, Tehran, 14115-111, Iran

**Keywords:** Biochemistry, Biological techniques, Cancer, Cell biology, Computational biology and bioinformatics, Genetics, Molecular biology

## Abstract

MicroRNA expression in breast cancer (BC) is explored both as a potential biomarker and for therapeutic purposes. Recent studies have revealed that miR-203a-3p is involved in BC, and importantly contributes to BC chemotherapy responses; however, the regulatory pathways of miR-203a in BC remain elusive. Hence, we aimed to investigate the miR-203a regulatory mechanisms and their potential functions in the progress of BC. To this end, the miR-203a potential involving pathways was predicted by databases analyzing its target genes. The relations between miR-203a, the phosphatidylinositol 3′-kinase (PI3K)–Akt, and Wnt signaling pathways were mechanistically investigated. Our results revealed that miR-203a inhibited the activation of the PI3K/Akt and Wnt pathways and reduced its downstream cell cycle signals, including Cyclin D1 and c-Myc. Moreover, the overexpression of miR-203a drastically arrested the cell cycle at subG1 and G1 phases, decreased the viability, proliferation, and migration, and increased apoptosis of BC cells. Therefore, miR-203a-3p may be considered a tumor suppressor factor and a potential biomarker or therapeutic target for BC.

## Introduction

Breast cancer (BC) is the most common type of cancer in females worldwide. It is still considered a major cause of death in women despite the decrease in mortality rates of this cancer around the world^1^.

The abnormal expression of miRs is associated with progression and metastasis in various human cancers^2^ including BC^3,4^. Therefore, miRs may consider potential biomarkers for the prediction, diagnosis, and prognosis of treatment efficacy in BC.

It has been reported that miR-203a-3p plays a tumor suppressor role in BC^5,6^, and may be served as a possible prognostic marker in BC^7,8^. Furthermore, miR-203a functions in resistance and response to drugs such as cisplatin^9^, doxorubicin^10^, and 5-fluorouracil^11^. Thus, miR-203a is a potential therapeutic target to overcome chemoresistance in BC, and the investigation of its molecular mechanism is therefore of great importance to improvement in chemotherapy efficacy. Previous studies showed that miR-203a suppresses cell proliferation and migration by targeting BIRC5 and LASP1 in triple-negative BC cells^12^. Moreover, miR-203a by regulating SNAI1 and SNAI2 plays a role in the epithelial-to-mesenchymal transition (EMT) and tumor metastasis of BC^13–15^. However, the targets, key pathways, and functions of miR-203a in regulating BC still are elusive.

This study, using bioinformatics and experimental pathways analysis of miR-203a targeted genes revealed that miR-203a inhibits PI3K/Akt and Wnt pathways by targeting PIK3CA and Wnt2b. Functional analysis suggested that miR-203a is a potential tumor suppressor that controls BC cell cycle, proliferation, apoptosis and migration through PI3K/Akt and Wnt pathways.

## Results

### MiR-203a inhibits the PI3K/Akt pathway in BC cells

**Figure 1:**
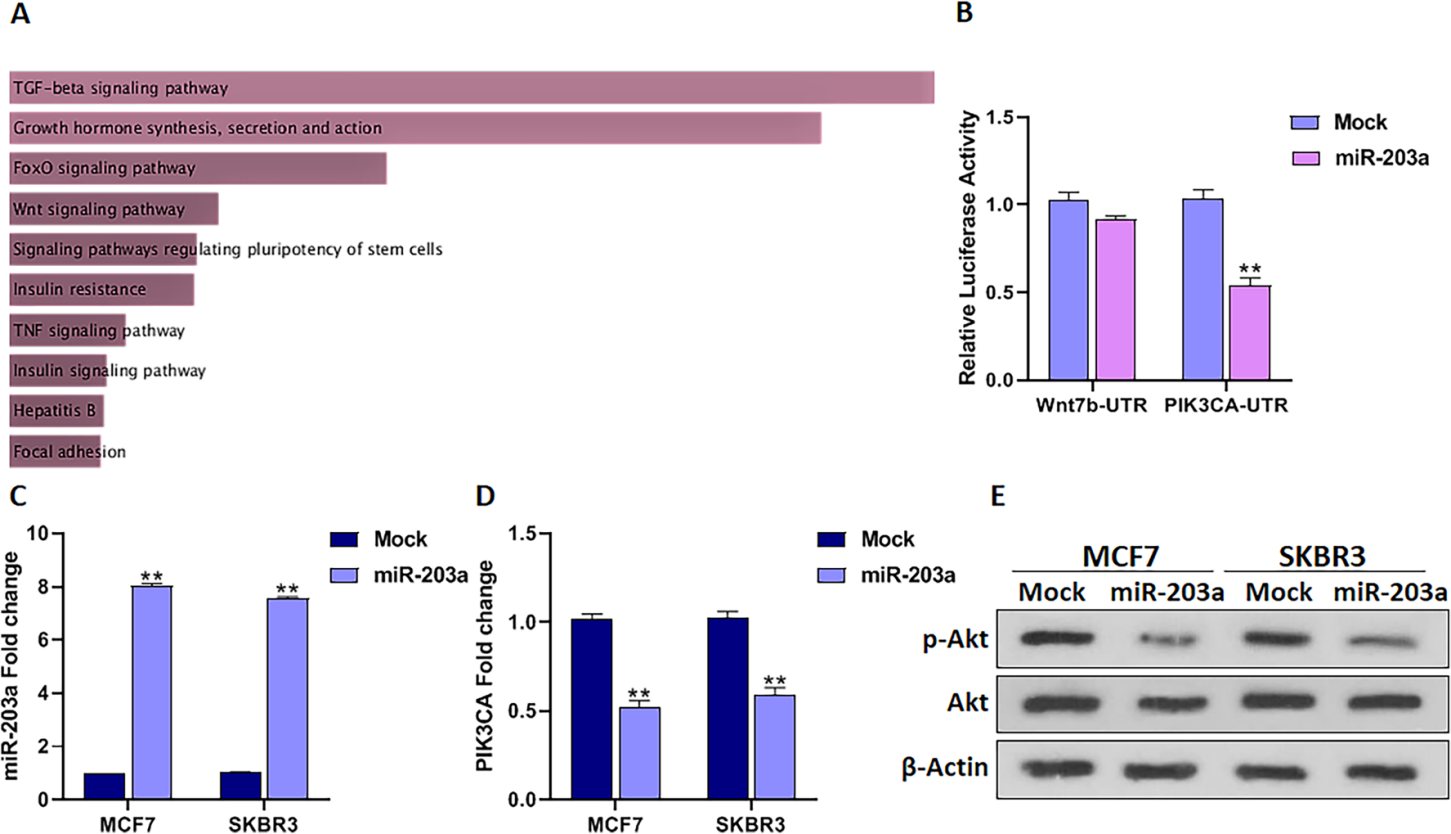
MiR-203a regulates PI3K/Akt signaling through its ability to target PIK3CA. (**A**) Flowchart of enriched KEGG pathways (www.kegg.jp/kegg/kegg1.html) of the genes correlated with miR-203a expression categorized according to their *P*-value. (**B**) PIK3CA is a direct target of miR-203a shown by 3′UTR luciferase assay. The 3′UTR sequence of Wnt-7b was used as off-target in this experiment. (**C**) The transfection efficiency of miR-203a was measured by qRT-PCR 48h after transfection (**D**) miR-203a upregulation lowered PIK3CA mRNA level as measured by the qRT-PCR method. (**D**) Western blot of p-Akt and Akt showed that miR-203a inhibited Akt phosphorylation in BC cells. *P*-values as described in Fig. 1.

To gain insight into the miR-203a involving pathways, computational miRNA target prediction tools, TargetScan, and miRDB were applied to identify miR-203a target genes (Table S1). Then, miR-203a target genes were categorized by pathway enrichment analysis, Kyoto Encyclopedia of Genes and Genomes (KEGG) pathway according to their *P*-value using the Enrichr website database. The KEGG pathway analysis is a useful way to get insight into the biological pathways of genes^16^. According to this analysis, miR-203a target genes are mainly involved in the transforming growth factor β (TGF-β) signaling pathway (Fig. 1A) which has been previously reported as miR-203a involving pathway in BC^14^. The Growth hormone synthesis, secretion, and action is the second highest and has not been studied pathway related to miR-203a in BC; therefore, we focused on this pathway.

PI3K/Akt pathway-related gene, *PIK3CA* which has previously been considered as miR-203a direct target gene^17,18^ is related to the Growth hormone pathway. To confirm whether *PIK3CA* is a direct target of miR-203a in BC, luciferase reporter constructs were integrated with the 3'UTR of PIK3CA comprising the miR-203a binding sites and 3'UTR sequence of Wnt-7b as an off-target control. The luciferase activity analysis showed that this activity was considerably reduced in the PIK3CA 3'UTR group transfecting with miR-203a compared to the off-target control group, Wnt-7b 3'UTR sequence construct (*P* < 0.01, Fig. 1B).

To further investigate, a plasmid-mediated overexpression of miR-203a was performed. The plasmid overexpressing miR-203a (miR-203a) and the same plasmid without miR-203a (Mock) were transfected to BC cells. The transfection efficiency was confirmed by qRT-PCR expression analysis. The plasmids were well-transfected and overexpressed in the MCF7 (*P* < 0.01) and SKBR3 (*P* < 0.01) cells (Fig. 1C).

To examine the impact of miR-203a PIK3CA targeting, the PIK3CA expression was measured in BC cells in miR-203a overexpressed condition compared to the control condition. The qRT-PCR results showed a significant decrease of PIK3CA levels in MCF7 (*P* < 0.01) and SKBR3 (*P* < 0.01) cells (Fig. 1D) after overexpression of miR-203a compared to the mock. Therefore, PI3KCA is a miR-203a direct target to modulate PI3K/Akt pathway in BC, as previously revealed that when miR-203a targets PIK3CA, it reduces PIK3CA mRNA and protein levels^18–21^. The activation of the PI3K/Akt plays an important role in BC development and PIK3CA mutations lead to PI3K/Akt activation^22^. It has been previously reported that miR-203a through targeting the 3'UTR sequence of PIK3CA reduces PIK3CA expression causing the inactivation of the PI3K/Akt signaling pathway in several cells^18–21^. To evaluate the possible role of miR-203a on Akt activation by targeting PI3KCA in BC, the phosphorylated Akt (p-Akt) and total Akt protein levels were measured. The p-Akt/Akt level decreased under overexpression of miR-203a by 0.58-fold in MCF7 cells and by 0.63-fold in SKBR3 cells compared to the mock (Fig. 1E). The other reported target gene of miR-203a from the Growth hormone pathway is *Akt3*^23^. Thus, the effect of miR-203a on the Akt3 expression was also assessed. However, there was no significant change in Akt3 level by miR-203a overexpression (Fig. S1). Thus, these results suggest that miR-203a modulates the PI3K/Akt signaling pathway by targeting PIK3CA. The Akt, as a serine/threonine kinase, is a vital component in the PI3K/Akt signaling cascade^24^, and deregulation of this pathway is known as a potential cause of treatment failure in BC^24,25^.

### MiR-203a inhibits the Wnt/β-Catenin pathway in BC cells

**Figure 2:**
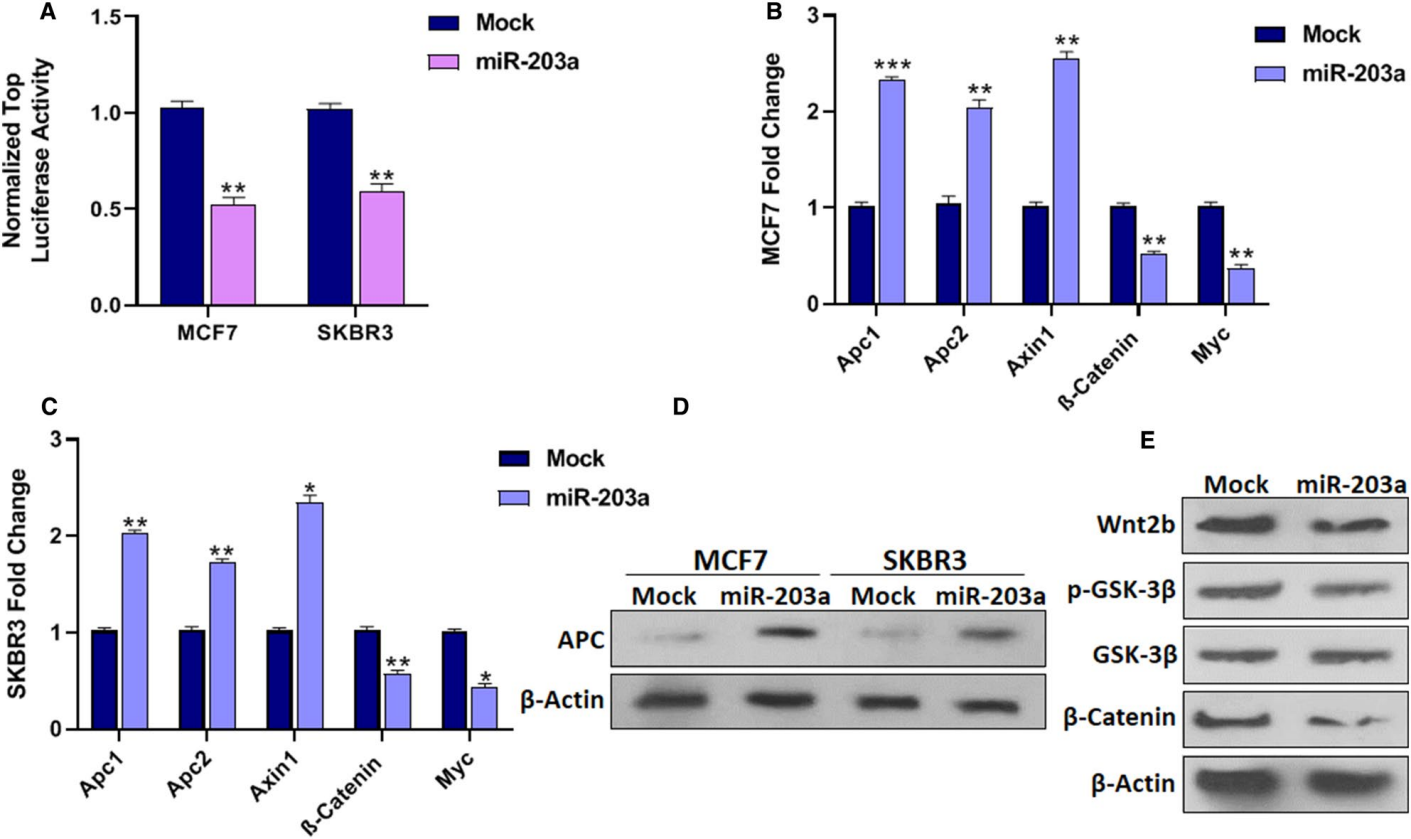
Association of miR-203a expression with Wnt signaling. (**A**) BC cells were co-transfected using TOP flash plasmid with either control or miR-203a expressing vectors. TOP flash luciferase activity indicated that overexpression of miR-203a was followed by significantly decreased Wnt activity in the BC cells. (**B**) miR-203a upregulation was followed by increased APC1, APC2, and Axin expression while decreased β-Catenin and c-Myc expression in MCF7 cells and (**C**) SKBR3 cells. (**D**) The western blot results confirmed the increased APC protein levels in BC cells by miR-203a overexpression. (**E**) Wnt2b, p-GSK-3β, and β-Catenin protein levels were decreased in MCF7 cells by overexpression of miR-203a as measured by Western blotting. *P < 0.05, **P < 0.01, ***P < 0.001.

According to KEGG enrichment analysis, miR-203a targeted genes also play a role in the Wnt pathway (Fig. 1A); however, this pathway has not been studied related to miR-203a in BC. Therefore, we also explored the relationship between miR-203a and the Wnt pathway. It has been known that the Wnt/β-Catenin pathway affects carcinogenesis and epithelial-to-mesenchymal transition (EMT) activation^26^. To evaluate the effect of miR-203a on the Wnt signaling pathway, different approaches were taken. Firstly, a TOP flash assay was performed to measure Wnt activity. As shown in (Fig. 2A), miR-203a overexpression significantly decreased the luciferase activity of the pGL3-TopFlash reporter in MCF7 (*P* < 0.01) and SKBR3 (*P* < 0.01) cells compared with control cells.

Secondly, the mRNA expression levels of Wnt-pathway-related genes were assessed. In MCF7 cells, the expression of adenomatous colonic polyposis colonic 1 (APC1) (*P* < 0.001), APC2 (*P* < 0.01), and axis inhibition 1 (Axin1) (*P* < 0.01) remarkably increased while the expression of β-Catenin (*P* < 0.01) and c-Myc (*P* < 0.01) highly decreased after miR-203a overexpression related to mock-transfected cells (Fig. 2B). Likewise, in SKBR3 cells, the expression of APC1 (*P* < 0.01), APC2 (*P* < 0.01), and Axin1 (*P* < 0.05) significantly increased while the expression of β-Catenin (*P* < 0.01) and c-Myc (*P* < 0.05) remarkably decreased when miR-203a overexpressed compared to mock-transfected cells (Fig. 2C). Furthermore, the expression levels of APC was also measured at the protein level. The western blotting results indicated that the APC level increased in MCF7 cells by 2.1-fold and in SKBR3 cells by 1.9-fold when miR-203a was overexpressed compared to the mock (Fig. 2D).

Thirdly, the TargetScan tool predicts that APC, Axin1, and Wnt2b are miR-203a target genes in the Wnt pathway. Because there was no decrease in the mRNA expression levels of APC and Axin1, Wnt2b was considered a possible direct target. Previously, Wnt2b has been reported as a direct miR-203a target gene^27^. Confirming this possibility, the protein level of Wnt2b decreased by 0.76-fold in miR-203a overexpression compared to mock-transfected MCF7 cells (Fig. 2E). However, the extent of alteration in Wnt2b protein level was not as significant as seen for its downstream APC and β-Catenin proteins. Therefore, miR-203a affects the Wnt/β-Catenin pathway also through another mechanism.

The Wnt pathway connects to the PI3K/Akt pathway through Glycogen synthase kinase-3β (GSK-3β), a serine/threonine protein kinase. GSK-3β, an important molecule downstream of Akt, is a negative regulator of the Wnt/β-Catenin cascade, along with Axin and APC^28^. Therefore, we assumed that miR203a also modulates the Wnt pathway through Akt/GSK-3β pathway. To evaluate this hypothesis, the phosphorylated GSK-3β (p-GSK-3β) and total GSK-3β protein levels were measured. The p-GSK-3β/GSK-3β level decreased when miR-203a overexpressed by 0.63-fold, compared to the mock (Fig. 2E). Additionally, the decreased expression of β-Catenin which is a downstream gene of GSK-3β and Wnt pathway was also confirmed at the protein level. The results demonstrated that miR-203a upregulation decreased β-Catenin protein level by 0.48-fold in MCF7 cells (Fig. 2E) compared with mock control. These approaches indicate that miR-203a regulates the Wnt pathway by targeting Wnt2b and through Akt/GSK-3β axis by targeting PIK3CA. Collectively, the results determined that miR-203a overexpression suppresses cancerous phenotypes of BC cells by repressing the PI3K/Akt and Wnt signaling pathways; thus, the influence of miR-203a on cancerous phenotypes of BC cells was assessed.

### MiR-203a inhibits the cell cycle and proliferation in BC cells

**Figure 3:**
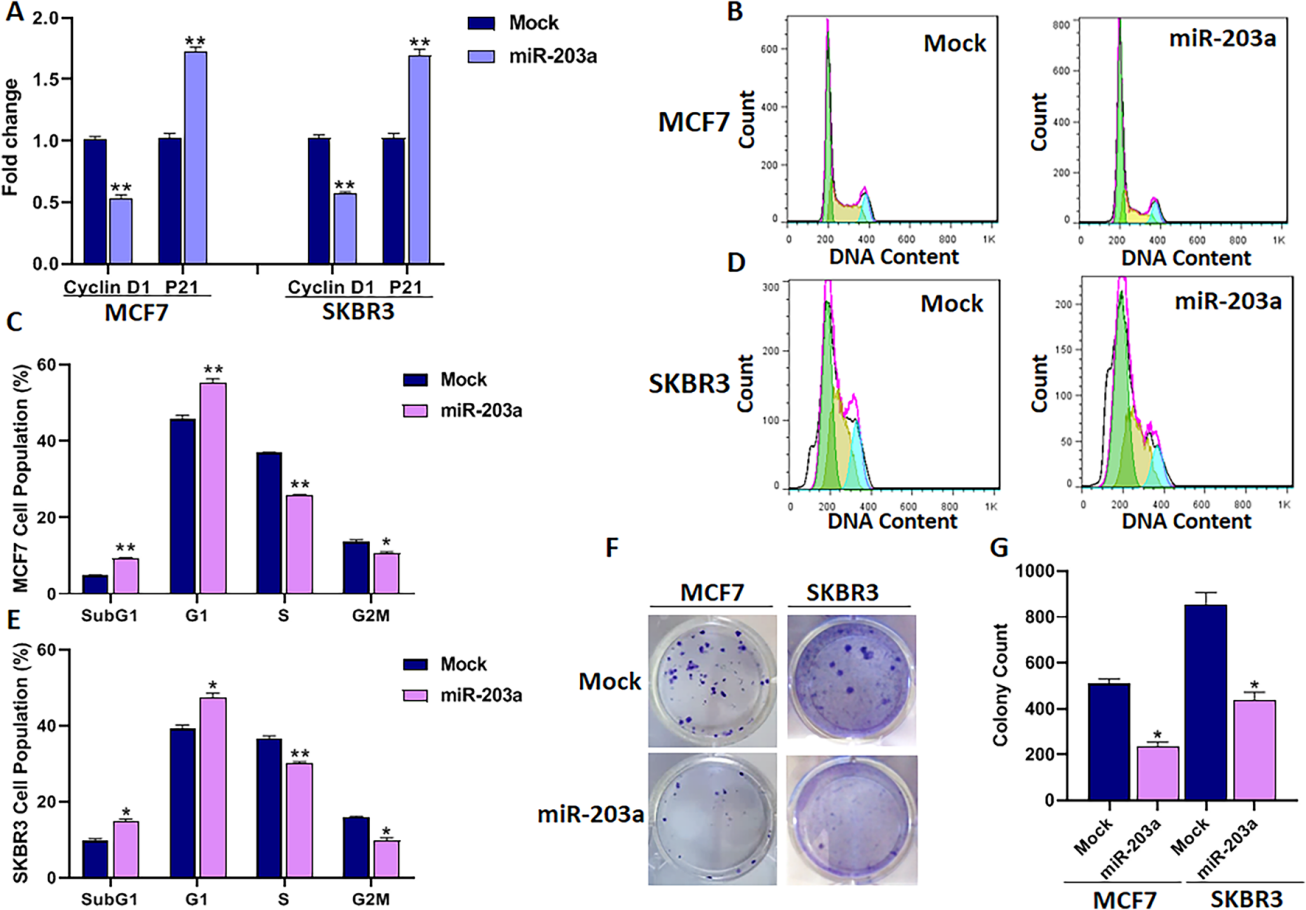
The effect of miR-203a overexpression on cell cycle and proliferation of BC cells. (**A**) Cyclin D1 and P21 mRNA levels. (**B**) Histogram analysis of MCF7 and (**D**) SKBR3 cells cycles transfected with miR-203a overexpressing vector or mock vector 48h after transfection. (**C**), and (**E**) Bar plot analysis of BC cells percentage in each phase of the cell cycle, following the transfection. (**F**) Colony formation assay following miR-203a overexpression (**G**) Bar plot analysis of colony formation. *P < 0.05, **P < 0.01.

To evaluate the impact of miR-203a on the cell cycle, the expression analysis of cell cycle-related genes was performed. Cyclin D1 is an essential regulator in the G1 phase and an oncogenic driver in cancer cells, and CDK inhibitor p21 mediates the cell cycle negatively. Besides, Cyclin D1 is a downstream target gene of both PI3K/Akt and Wnt pathways. The qRT-PCR indicated that overexpression of miR-203a in MCF7 and SKBR3 cells resulted in the decrease of Cyclin D1 (cell cycle activator) (*P* < 0.01) and the increase of P21 (cell cycle inhibitors) (*P* < 0.01) levels compared to mock-transfected cells (Fig. 3A). In addition, western blot analysis demonstrated that Cyclin D1 was also decreased at protein level by 0.4-fold in MCF7 cells when miR-203a overexpression (Fig. S2).

Next, the cell cycle analysis demonstrated that MCF7 cells overexpressing miR-203a increased the sub-G1 (*P* < 0.01) and G1 (*P* < 0.01) cell proportions and decreased the S (*P* < 0.01) and G2/M (*P* < 0.05) cell proportions compared to mock-transfected cells (Fig. 3B,C). Similarly, the SKBR3 cells with overexpressed miR-203a revealed higher the sub-G1 (*P* < 0.01) and G1 (*P* < 0.01) cell proportions and reduced the S (*P* < 0.01) and G2/M (*P* < 0.05) cell proportions related to cells with mock-transfected (Fig. 3D,E).

The possible influence of miR-203a on BC cell proliferation and viability was evaluated using colony formation and MTT assays. The colony-formation assay revealed that miR-203a overexpression significantly decreased colony formation of MCF7 (*P* < 0.05) and SKBR3 (*P* < 0.05) cells compared to mock-transfected cells (Fig. 3F,G). The MTT assay showed that miR-203a overexpression significantly inhibited the viability of MCF7 (*P* < 0.05) and SKBR3 (*P* < 0.05) cells at 72 h after transfection (Fig. S3). These results indicate that miR-203a inhibits the cell cycle, proliferation, and viability in BC cells via PI3K/Akt and Wnt pathways because Cyclin D1 is the downstream gene of these two pathways and both are involved in the proliferation and survival.

### MiR-203a enhances apoptosis in BC cells

**Figure 4:**
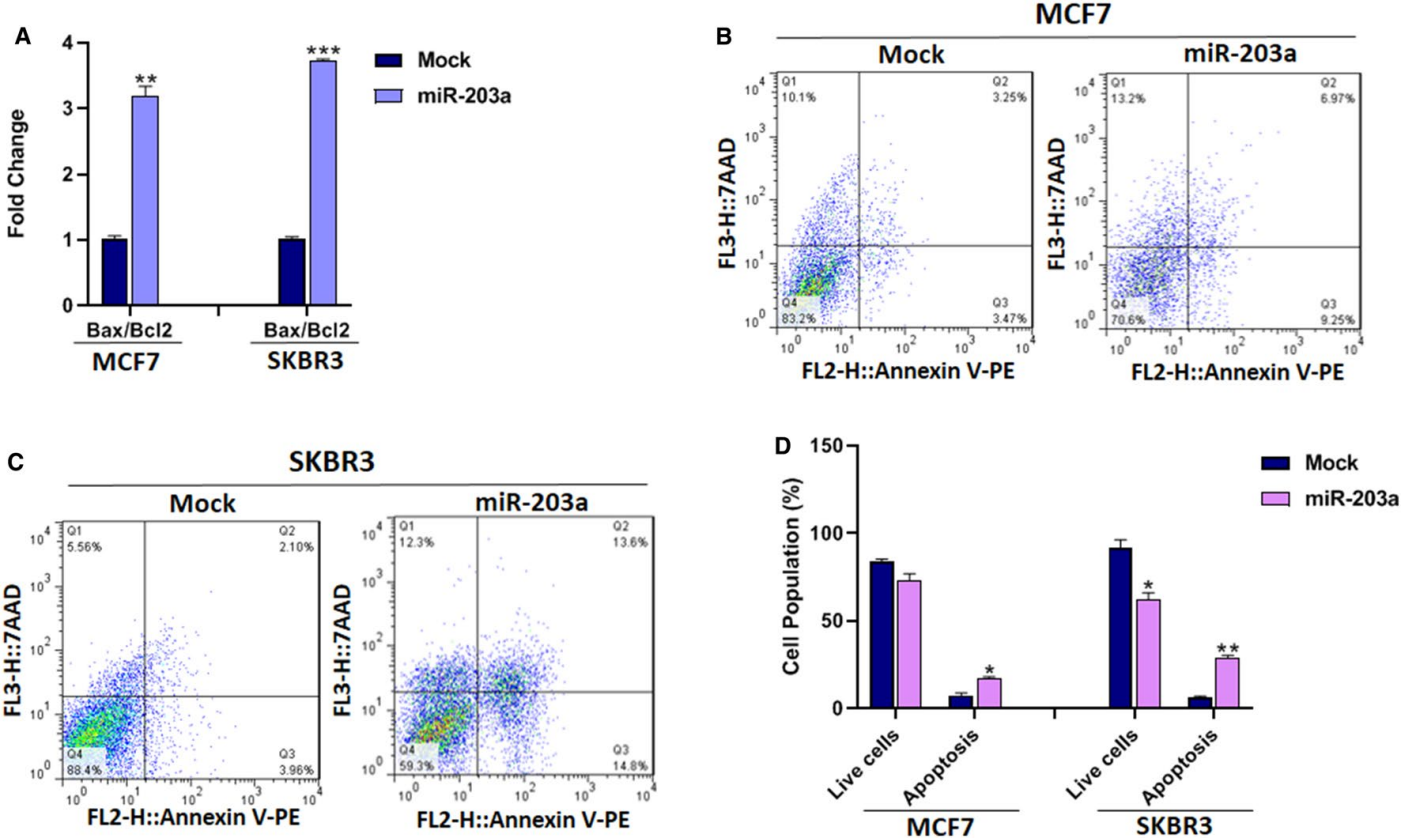
The effect of miR-203a overexpression on apoptosis of BC cells. (**A**) The ratio of mRNA expression of Bax to Bcl2 in BC cells with or without miR-203a overexpression. (**B** and **C**) Represents Annexin flow cytometry dot plots and (**G**) bar plot of BC cells percentage in different phases. *P < 0.05, **P < 0.01, ***P < 0.001.

To evaluate the influence of miR-203a on cell apoptosis, the expression analysis of apoptosis-related genes was investigated. The mRNA expression analysis showed an increase of Bax (apoptotic marker)/Bcl2 (anti-apoptotic marker) ratio in MCF7 (*P* < 0.01) and SKBR3 (*P* < 0.001) when miR-203a was overexpressed (Fig. 4A).

The role of miR-203a on the apoptosis of BC cells was also measured by PI/Annexin V assay. The result of the flow cytometry assay indicated that in the MCF7 and SKBR3 cells overexpressing miR-203a, the apoptotic (early and late) cell percentages were significantly higher than mock-transfected cells (*P* < 0.05) and (*P* < 0.01) respectively (Fig. 4B–D). These results indicate that miR-203a promotes apoptosis in BC cells, thus lowered proliferation of miR-203a overexpressed cells may be a consequence of apoptosis.

### MiR-203a inhibits the migration in BC cells

The Wnt/β-Catenin pathway is involved in the EMT program and its consequence is cell migration and invasion as characteristics of metastatic cancer cells^26^_._ The β-Catenin which is regulated by miR-203a is also a mesenchymal marker that plays a role in EMT processes. To this end, the influence of miR-203a on the EMT process and migration was assessed through different approaches.

**Figure 5:**
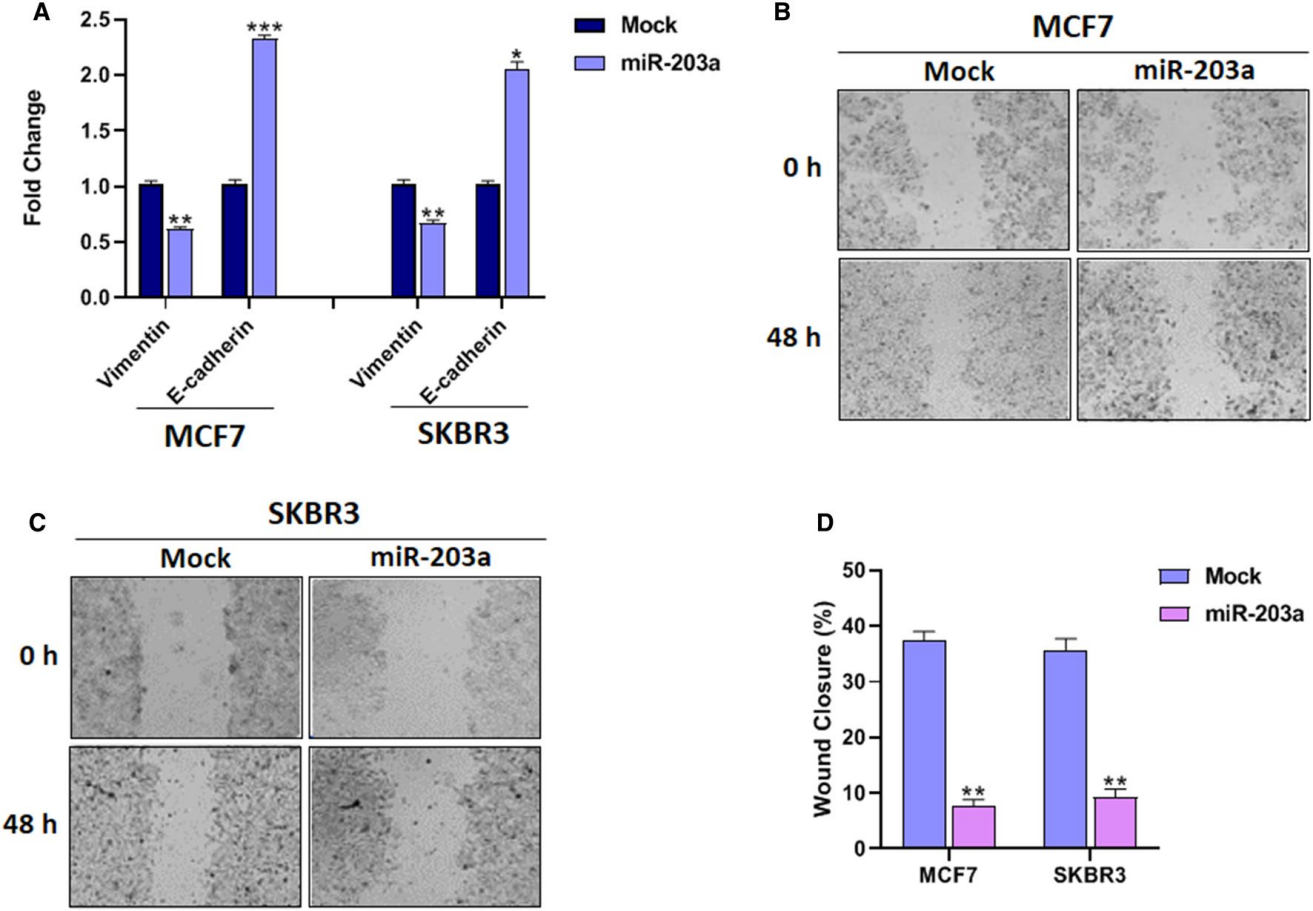
The effect of miR-203a overexpression on migration BC cells. (**A**) qRT-PCR analysis of epithelial and mesenchymal markers in BC cells with or without miR-203a overexpression. (**B**) Wound healing analysis of MCF7 and (**C**) SKBR3 cells transfected with either miR-203a or mock vector at 0 and 48h post-scratching, Scale bar = 200 μm, (**D**) Quantitative analysis of scratch wound closure.*P < 0.05, **P < 0.01, ***P < 0.001.

Firstly, the mRNA expression levels of E-cadherin and Vimentin, two important EMT marker genes were assessed in BC cells with or without miR-203a overexpression. The expression of E-cadherin (epithelial marker) drastically increased in MCF7 (*P* < 0.01) and SKBR3 (*P* < 0.01) cells while the expression of Vimentin (mesenchymal marker) significantly decreased in MCF7 (*P* < 0.001) and SKBR3 (*P* < 0.01) cells in miR203a overexpression relative to mock control (Fig. 5A).

Secondly, performing wound healing assay cells migration at different time points after scratching was monitored under a microscope. The result indicated that the wound closure rate in BC cells expressing miR-203a lowered at 48h relative to mock-transfected cells, MCF7 cells (*P* < 0.05), and SKBR3 cells (*P* < 0.05) (Fig. 5B–D). Taken together, these results suggest that miR-203a suppresses the EMT process and cell migration in BC cells.

## Discussion

Previous studies on miR-203a have reported BIRC5, LASP1, and SNAI2 as miR-203a targets in mediating the development of BC^13,16^. Moreover, miR-203a is controlled by TGF-β via SNAI2 to modulate the EMT process in BC^15^. However, there might be other mechanisms that contribute to BC progression by miR-203a that have not yet been identified. It has been previously revealed that miR-203a directly targets both PIK3CA and Wnt2b reducing these genes' expression at mRNA and protein levels in other cells^19–21,27^. This study also confirmed PIK3CA as a direct target of miR-203a mediating the Akt activation in BC cells. Moreover, miR-203a modulates the Wnt pathway by Wnt2 targeting and through Akt/GSK-3β axis via PIK3CA targeting. Thus, miR-203a inhibits cell cycle, proliferation, and migration.

The Akt activation inhibits GSK-3β; thus, the β-Catenin destruction complex which consists of APC, GSK-3β, AXIN, CK1, PP2A, and β-trcp unable to bind and degrades β-Catenin. Consequently, β-Catenin accumulates and translocates to the nucleus. When β-Catenin gets translocated to the nucleus, acts as a transcriptional factor to stimulate the expression of downstream target genes such as Cyclin D1, c-Myc, MMP14, Slug, FN1, C-JUN, L1CAM, and CD44^29^. These genes are related to various oncogenic pathways. Besides, Cyclin D1 and c-Myc are cell cycle factors to promote cell cycle progression and proliferation in cancers^30^, consisting of BC^31^.

**Figure 6:**
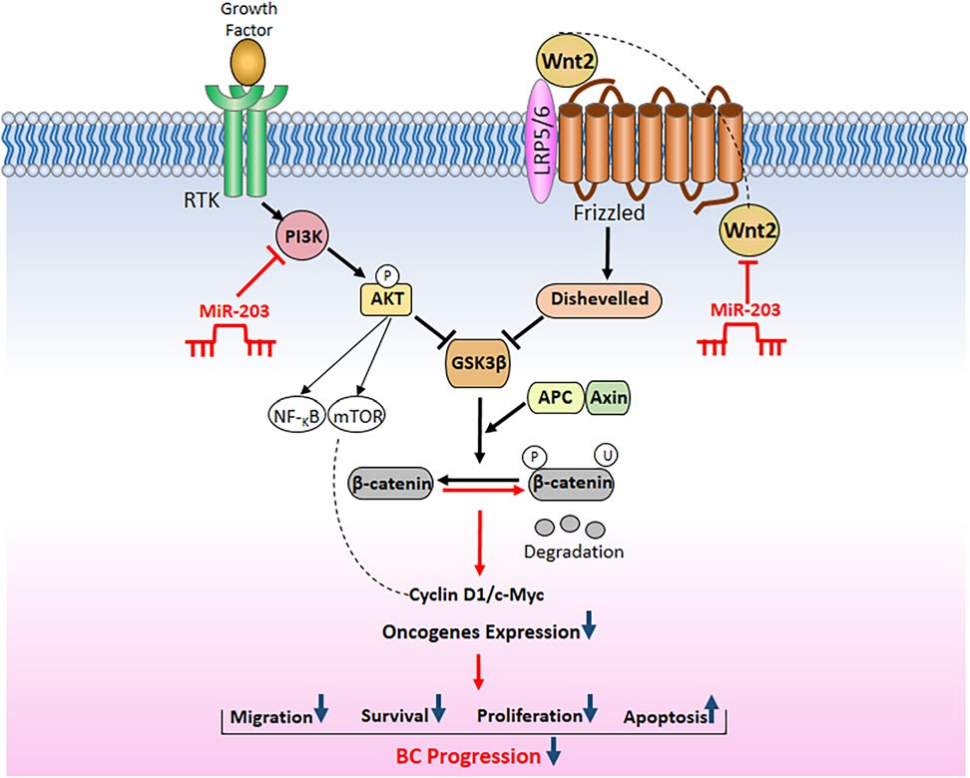
A schematic representative of the molecular mechanisms by which miR-203a affects cancerous phenotypes of BC cells through the PI3K/Akt and Wnt signaling pathways.

Akt also has other target proteins rather than GSK for the proliferation modulation such as mTOR, NF-_Κ_B, BAD, and FoxO1^32^. Thus, the influence of miR-203a on proliferation inhibition through Akt inactivation could be via other proteins rather than GSK-3β. Additionally, GSK-3β could be regulated by other Wnt/β-Catenin signaling components. When the Wnt/β-Catenin pathway is activated, the Dvl2 (Disheveled 2) protein is up-regulated and GSK-3β is inhibited by phosphorylation, thereby decreasing β-Catenin degradation and accumulating β-Catenin in the cytosol. To this end, this study also aimed to assess the impact of miR-203a on Wnt/β-Catenin pathway genes. Bioinformatic analysis using TargetScan predicts APC and Axin1 as miR-203a targets. However, according to qRT-PCR results, APC and Axin1 were not miR-203a target genes in BC due to increased mRNA expression levels. However, the protein expression level of Wnt2b decreased. Taken together, as outlined in (Fig. 6), we explored the link between miR-203a and PI3K/Akt and Wnt pathways. MiR-203a overexpression reduces the inhibitory effects of Akt and Dishevelled proteins on GSK-3β activity by targeting PIK3CA and Wnt2b. Therefore, active GSK-3β binds to the β-Catenin destruction complex and degrades β-Catenin. Consequently, β-Catenin is unable to translocate to the nucleus and stimulate the expression of downstream target genes involved in cancerous phenotypes. The PI3K/Akt and Wnt/β-Catenin pathways are activated in several types of cancers^33^. The abnormal activation of the Akt/GSK3β/β-Catenin signaling pathway plays a key role in cancerous progress^34,35^.

In summary, this study indicates that the miR-203a serves as a tumor suppressor by regulating the PI3K/Akt and Wnt signaling pathways. MiR-203a upregulation inhibits malignant phenotypes of BC cells by reduction of PIK3CA expression and then, Akt inactivation. Furthermore, miR-203a modulates the Wnt pathway by Wnt2b targeting and through Akt/GSK-3β axis. Therefore, these results provide valuable insights into the clinical use of miR-203a in reducing the malignant phenotypes in BC.

## Materials and methods

### Plasmid construction

The sequence of the miR-203a precursor was amplified by PCR, TA-cloned into pGEM-T Easy Vector Systems (Promega), and then sub-cloned into a peGFP-C1 expression vector under CMV Promoter. The sequence was validated by performing Sanger sequencing. The sequences of primers used for the PCR reaction are in (Table 1).

**Table 1:** Primers were used in this study.

Gene	Forward primer (5′-3′)	Reverse primer (5′-3′)
miR-203a-3p	CGTTGTGAAATGTTTAGGACCAC	
snRNA-U48	TGATGACCCCAGGTAACTCTG	
Universal		GCGTCGACTAGTACAACTCAAG
Pre-miR-203	CGCGACCAGCGGGGATCTG	CATGGGGCGGCCGACCT
PIK3CA	GGTGAAAGACGATGGACAACTGT	TGTAACACATCTCCTGAAACCTCTC
PIK3CA Cloning	CTCGAGAAAGATAACTGAGAAAATGAAAGCTC	TCTAGACCTTCTCCATCATTTCTATATATTTTGG
APC-1	TATTACGGAATGTGTCCAGCTTG	CCACATGCATTACTGACTATTGTC
APC-2	CGCACCCGTGAGGACTACAGGC	GATCATCTTGTGCTTGGAGTGCACC
Axin1	ATGCAGGAGAGCGTGCAGGTC	TGACGATGGATCGCCGTCCTC
β-catenin	AGAACAGAGCCAATGGCTTG	CCTGGCCATATCCACCAGAG
C-Myc	CTCCTACGTTGCGGTCACAC	CGGGTCGCAGATGAAACTCT
Cyclin D1	CAATGACCCCGCACGATTTC	CATGGAGGGCGGATTGGAA
P21	CACTCCAAACGCCGGCTGATCTTC	TGTAGAGCGGGCCTTTGAGGCCCTC
Vimentin	TTCCGTTCAAGGTCAAGACG	CGAGAGAAATTGCAGGAGGAG
E-cadherin	TGATATGAGGCTGTGGGTTCC	GACAGAGAAGACGCTGAGCAT
β-Actin	AGCACAGAGCCTCGCCTT	CATCATCCATGGTGAGCTGG

### Dual luciferase assay

In HEK293T cells, miR-203 overexpressed or negative mock control were transfected with one of the reporter vectors. Cells were collected at 48 h post-transfection and, a dual-luciferase reporter assay kit (Promega) was used to measure luciferase activity with a chemiluminescence meter.

### Cell culture and transfection

BC cells (Pasteur Institute of Iran) were cultured in DMEM containing (10%) FBS and (1%) penicillin–streptomycin in 5% CO2 at 37 °C. Cells were transfected using Lipofectamine 3000 (Invitrogen) according to the manufacturer's instructions. The cells were collected at indicated time points for the following experiments.

### QRT-PCR analysis

Total RNA was extracted using RiboEX (GeneAll) reagent, and used for RT reactions and qRT-PCR, according to the manufacturer's protocol (Takara). The expression of miR-203a and mRNAs was measured using SYBR Green reagent (AMPLIQON). Relative expression was assessed using the 2^−ΔΔCt^ method and normalized to U48/Actin (see (Table 1) for primers used in the study).

### Immunoblotting analysis

Total protein was extracted from cells using RIPA buffer and protein concentrations were determined with a Bio-Rad protein assay (Bio-Rad Laboratories). Equal amounts of protein were separated by SDS–polyacrylamide gel electrophoresis and electro blotted onto a polyvinylidene difluoride (PVDF) membrane. Membranes were blocked and labeled with antibodies targeting phospho-AKT (Elabscience), AKT (Elabscience), Wnt-2b, phospho-GSK3β, GSK3, APC, and β-Catenin, and Cyclin D1 (all purchased from Santa Cruz Biotechnology) and secondary HRP-conjugated antibodies. Actin B (Santa Cruz Biotechnology) was probed as a loading control. Proteins were detected by ECL (Thermo Fisher Scientific).

### TOP/FOP flash assay

The cells were seeded into the 24-well plates at 4 × 10^4^ cells/well and were transfected with (0.5 μg) of TOP flash vector along with (1 μg) of miR-203a overexpressing vectors. The cells were collected 48 h post transfection, and cell lysate was used for the luciferase activities measurement using a Luciferase reporter assay kit (Promega) and Luminometer.

### Cell cycle assay

Cells were transfected with the constructs and collected after 48 h and then stained with propidium iodide (PI, Sigma) for 30 min at room temperature in the dark. Next,

The number of cells in each cell cycle phase was defined by the FACSCalibur Flow Cytometry System (BD Bioscience).

### MTT assay

Cell viability was assessed by MTT assay. Briefly, 4 × 10^3^ cells/well were seeded into a 96-well plate and were incubated for 24 h. Then, at 24, 48, and 72 h after transfection, (10 μl) of MTT solution (5 mg/mL, Sigma-Aldrich) was added to each well. After incubation for another 4 h at 37 °C, the medium was discarded and (100 μl) of dimethyl sulfoxide (DMSO, Merck) was added to each well, and the plates were incubated for 15 min at 37 °C. The absorbance was measured by a micro plate scanning spectrophotometer (BioTeck) at 490 nm.

### Clone formation assay

MCF7 cells at a density of 1000 cells per well were assessed for colony formation after approximately 10 days with (1%) crystal violet staining.

### Apoptosis assay

BC cells were cultured (4 × 10^4^ cells/well) in 24-well plates, and 24 h after seeding cells were transfected with the constructs. 36 h after transfection, the cells were collected and stained using the Annexin-V-FITC/PI Staining Kit (Roche) and incubated for 15 min at room temperature in the dark. The cells were then evaluated by obtaining the PI- and Annexin V positive cells using a FACSCalibur Flow Cytometry System (BD Bioscience).

### Wound-healing assay

To assess the effect of the miR-203a on migration, MCF7 cells were seeded 4 × 10^4^ cells/well in a 24-well plate and incubated for 24h. After transfection, a 10 μl pipette tip was used to scratch the cells. The migration of cells toward the wound was captured under a microscope at different time points. The gap closure percent was measured using Image J software.

### Statistical analyses

Statistical analysis was performed using Graph Pad v.8.4.3. The data of all experiments was an average of two or three autonomous repeats. The results were expressed as mean ± standard deviation (SD). Student's two-tailed t-test and one-way ANOVA were performed to determine significance. The differences were considered significant when *P*-values < 0.05.

### Supplementary Information

Supplementary Information 1.

Supplementary Information 2.

Supplementary Information 3.

## Data Availability

The datasets analyzed during the current study are available in the (TargetScanHuman)/ https://www.targetscan.org/cgi-bin/targetscan/vert_80/targetscan.cgi?species=Human&mir_sc=miR-203a-3p.1, (Enrichr)/ https://maayanlab.cloud/Enrichr/, and (KEGG pathway)/ www.kegg.jp/kegg/kegg1.html.
