# Supplementary material for: MicroRNA-203a inhibits breast cancer progression through the PI3K/Akt and Wnt pathways
Source: Sci Rep. 2024 Feb 27;14:4715. doi: 10.1038/s41598-024-52940-5 (PMC10899204; doi:10.1038/s41598-024-52940-5)
Supplement: Supplementary file 1 — Supplementary Information 1. [file 41598_2024_52940_MOESM1_ESM.docx]

**MicroRNA-203a inhibits breast cancer progression through the** [**PI3K/Akt**](https://pubmed.ncbi.nlm.nih.gov/34981299/) **and Wnt pathways**

Maryam Entezari, Bahram M. Soltani, Majid Sadeghizadeh*

*Department of Molecular Genetics, Faculty of Biological Sciences, Tarbiat Modares University, Tehran 14115-111, Iran*

Correspondence: Majid Sadeghizadeh, Department of Molecular Genetics, Faculty of Biological Sciences, Tarbiat Modares University, Tehran 14115-111, Iran. E-mail: [Sadeghma@modares.ac.ir](mailto:Sadeghma@modares.ac.ir)

**S Figure 1.** MiR-203a upregulation did not affect Akt3 mRNA level as measured by the qRT-PCR method.

**Mock**

**miR-203a**


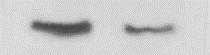


**Cyclin D1**


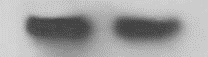


**β-Actin**

**S Figure 2.** The western blotting of Cyclin D1 protein level in MCF7 cells overexpressing miR-203a *vs.* control cells.

**S Figure 3.** The effect of miR-203a overexpression on the viability of BC cells. MTT assay monitored the survival rate of BC cells 72 hours post-transfection with miR-203a or mock vectors.
