# Supplementary material for: MicroRNA-203a inhibits breast cancer progression through the PI3K/Akt and Wnt pathways
Source: Sci Rep. 2024 Feb 27;14:4715. doi: 10.1038/s41598-024-52940-5 (PMC10899204; doi:10.1038/s41598-024-52940-5)
Supplement: Supplementary file 2 — Supplementary Information 2. [file 41598_2024_52940_MOESM2_ESM.docx]

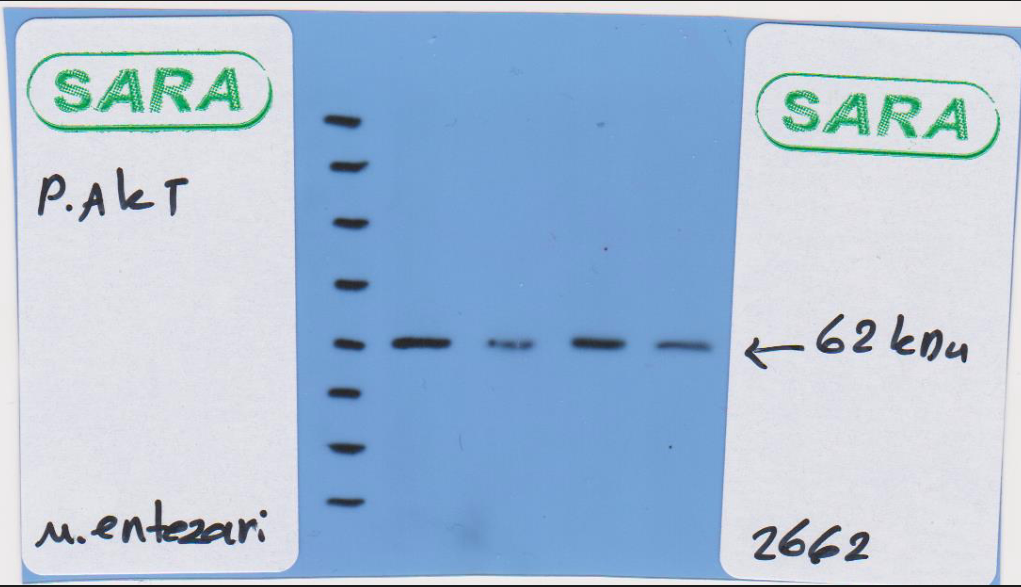


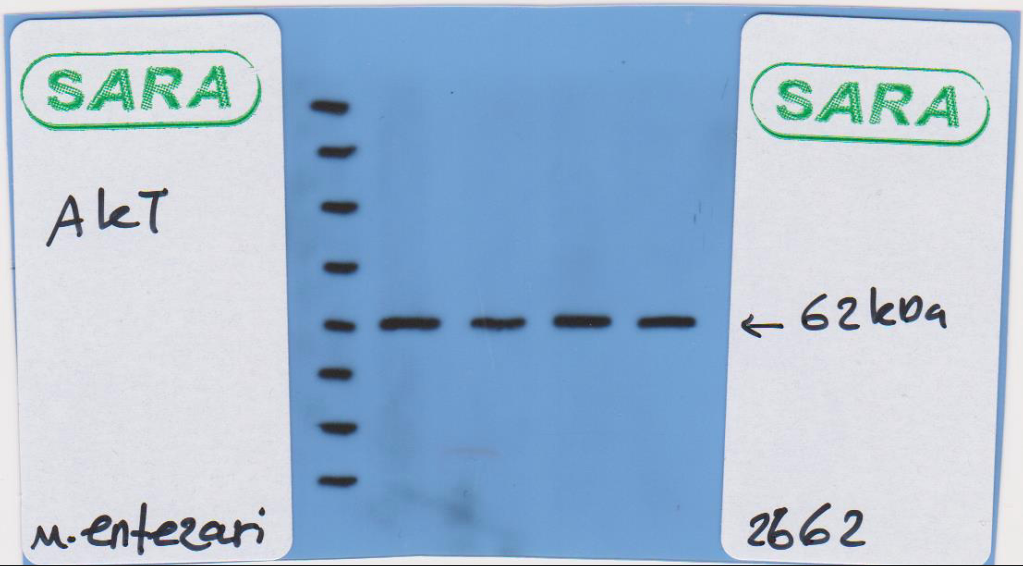


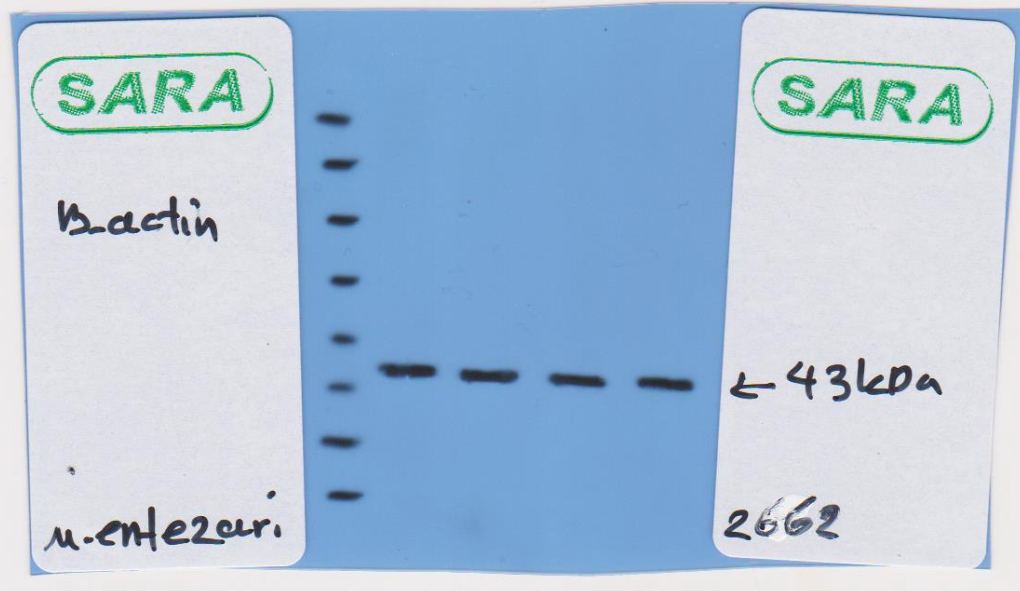


**Figure 1E.** Western blotting of the **Akt phosphorylation**. Lane1: protein molecular weight marker, Lane2: Mock in MCF-7, Lane3: miR-203a overexpression in MCF-7, Lane4: Mock in SKBR3, Lane5: miR-203a overexpression in SKBR3.


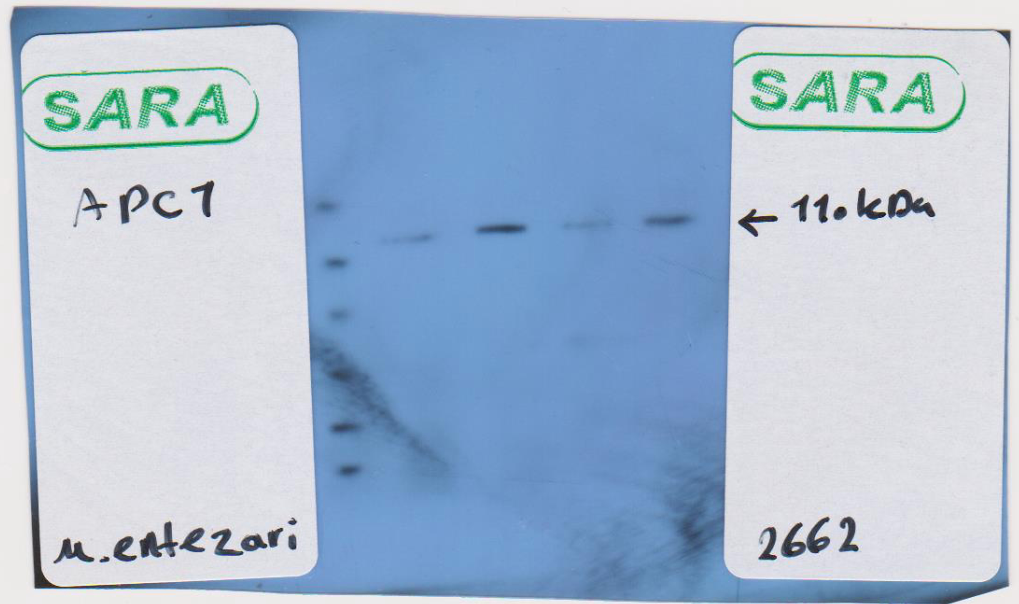


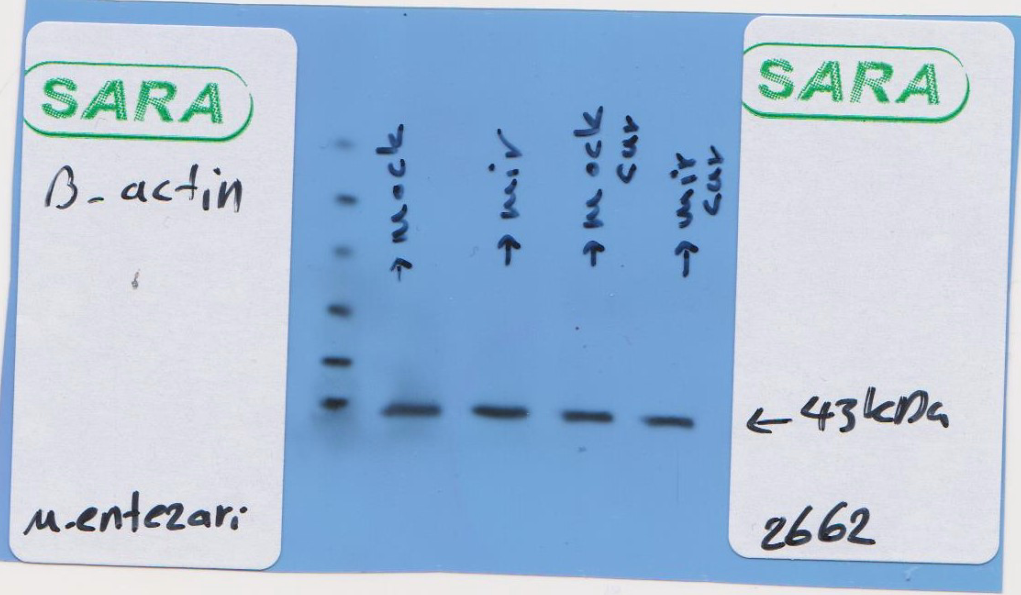


**Figure 2D.** Western blotting of the **APC**. Lane1: protein molecular weight marker, Lane2: Mock in MCF-7, Lane3: miR-203a overexpression in MCF-7, Lane4: Mock in SKBR3, Lane5: miR-203a overexpression in SKBR3.


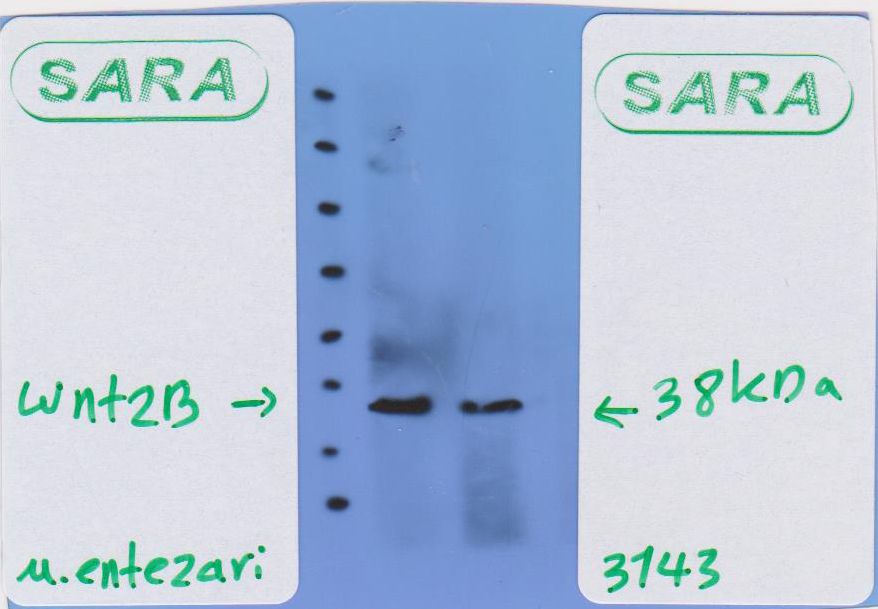


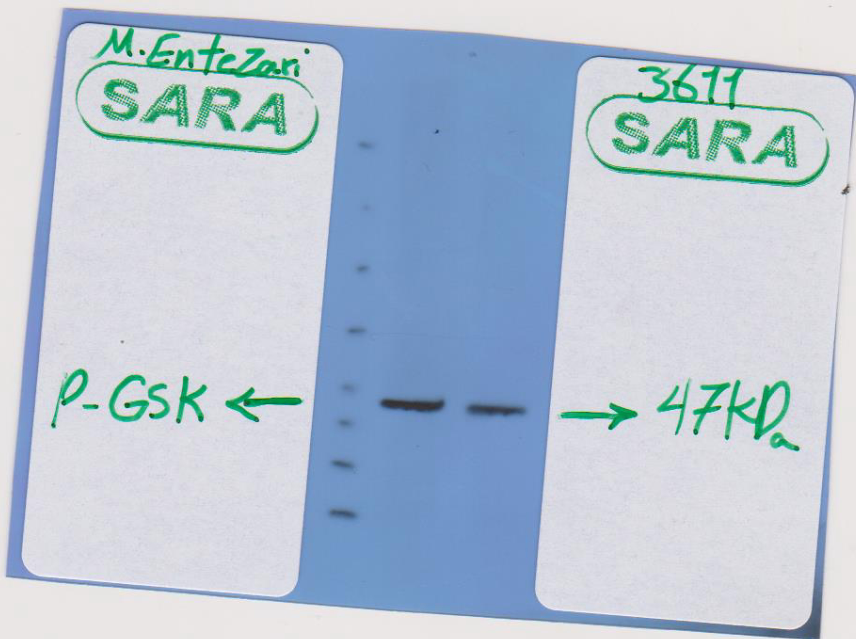


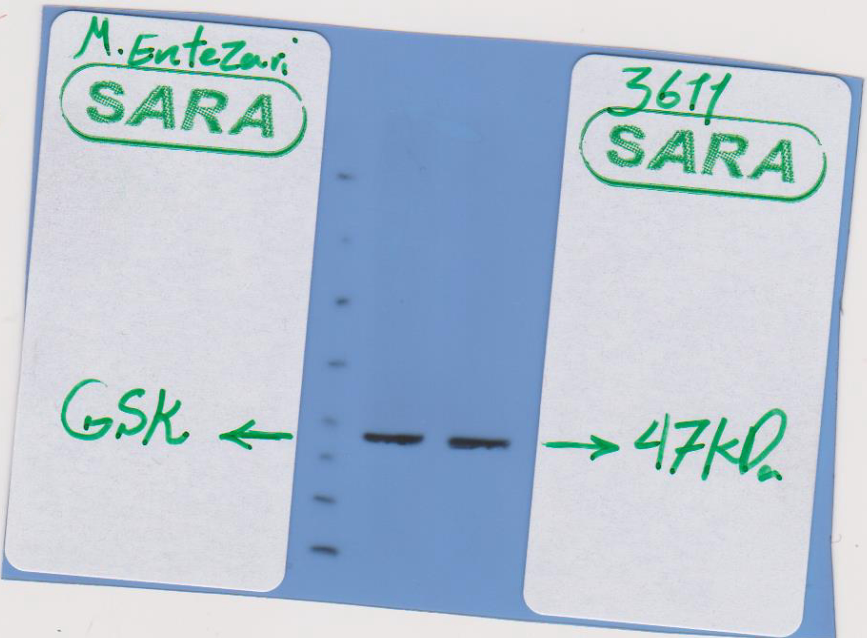


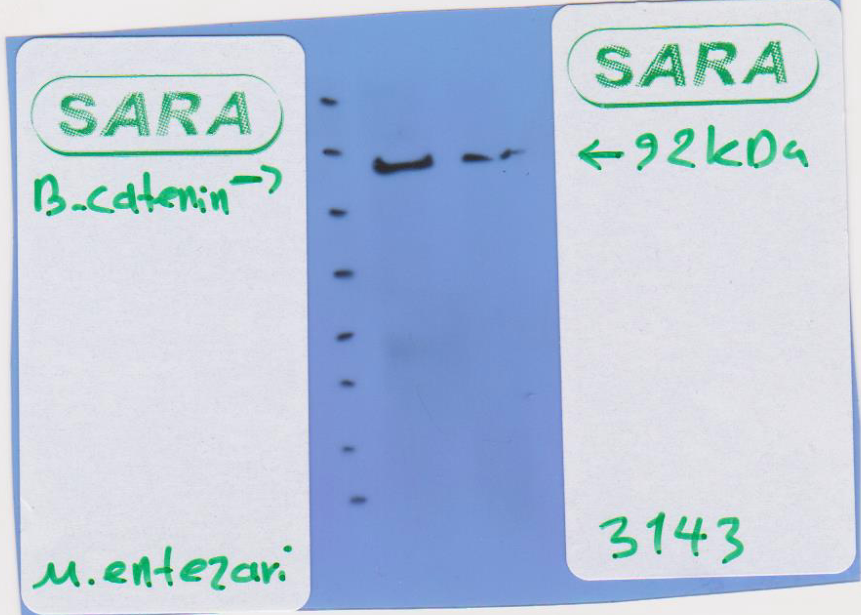


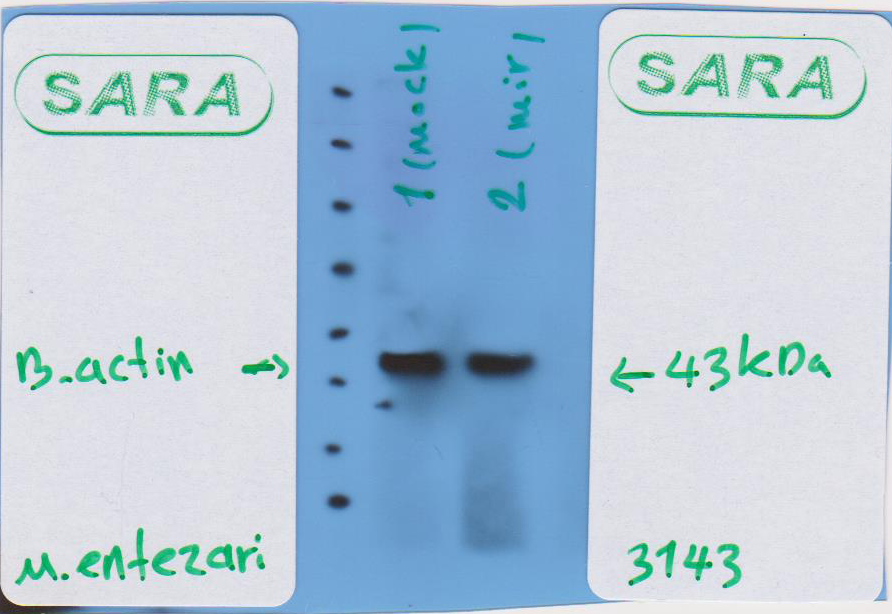


**Figure 2E.** Western blotting of the **Wnt2b, p-GSK, GSK and β-Catenin**. Lane1: protein molecular weight marker, Lane2: Mock in MCF-7, Lane3: miR-203a overexpression in MCF-7, Lane4: Mock in SKBR3, Lane5: miR-203a overexpression in SKBR3.

**
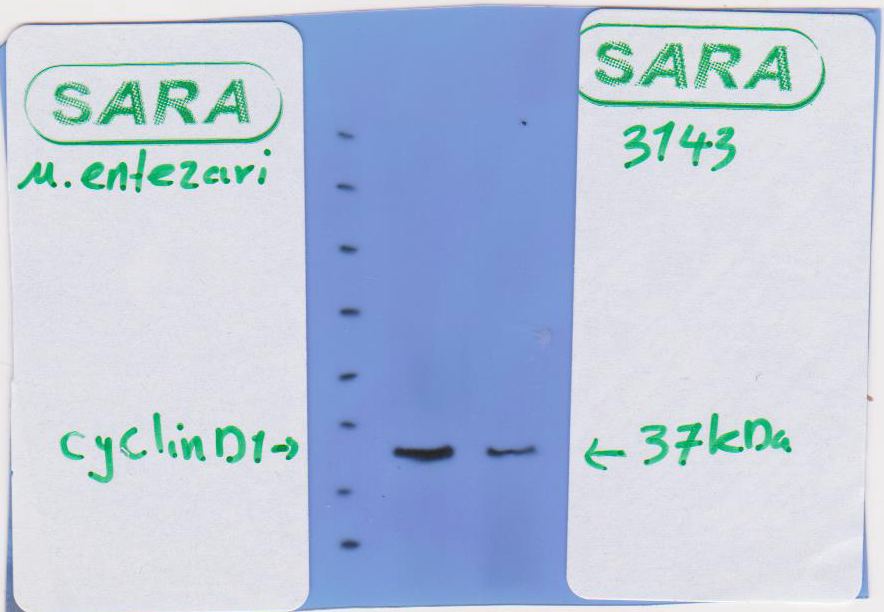
**

**
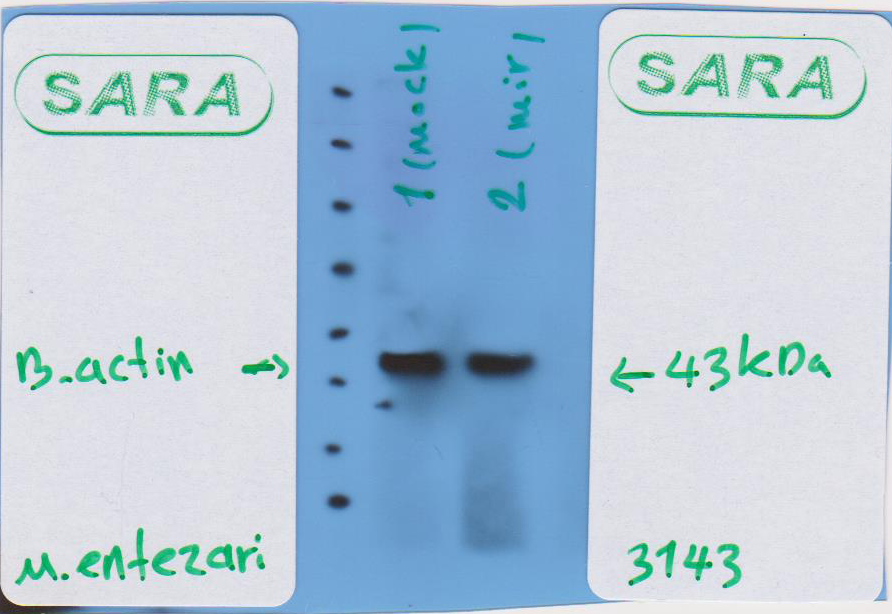
**

**S Figure 2.** Western blotting of the **Cyclin D1**. Lane1: protein molecular weight marker, Lane2: Mock in MCF-7, Lane3: miR-203a overexpression in MCF-7, Lane4: Mock in SKBR3, Lane5: miR-203a overexpression in SKBR3.
