# Supplementary material for: MicroRNA-203a inhibits breast cancer progression through the PI3K/Akt and Wnt pathways
Source: Sci Rep. 2024 Feb 27;14:4715. doi: 10.1038/s41598-024-52940-5 (PMC10899204; doi:10.1038/s41598-024-52940-5)
Supplement: Supplementary file 3 — Supplementary Information 3. [file 41598_2024_52940_MOESM3_ESM.docx]

| **Gene** | **Forward primer (5'-3')** | **Reverse primer (5'-3')** |
| --- | --- | --- |
| miR-203a-3p | CGTTGTGAAATGTTTAGGACCAC |  |
| snRNA-U48 | TGATGACCCCAGGTAACTCTG |  |
| Universal |  | GCGTCGACTAGTACAACTCAAG |
| Pre-miR-203 | CGCGACCAGCGGGGATCTG | CATGGGGCGGCCGACCT |
| PIK3CA | GGTGAAAGACGATGGACAACTGT | TGTAACACATCTCCTGAAACCTCTC |
| APC-1 | TATTACGGAATGTGTCCAGCTTG | CCACATGCATTACTGACTATTGTC |
| APC-2 | CGCACCCGTGAGGACTACAGGC | GATCATCTTGTGCTTGGAGTGCACC |
| Axin1 | ATGCAGGAGAGCGTGCAGGTC | TGACGATGGATCGCCGTCCTC |
| β-catenin | AGAACAGAGCCAATGGCTTG | CCTGGCCATATCCACCAGAG |
| C-Myc | CTCCTACGTTGCGGTCACAC | CGGGTCGCAGATGAAACTCT |
| Cyclin D1 | CAATGACCCCGCACGATTTC | CATGGAGGGCGGATTGGAA |
| P21 | CACTCCAAACGCCGGCTGATCTTC | TGTAGAGCGGGCCTTTGAGGCCCTC |
| Vimentin | TTCCGTTCAAGGTCAAGACG | CGAGAGAAATTGCAGGAGGAG |
| E-cadherin | TGATATGAGGCTGTGGGTTCC | GACAGAGAAGACGCTGAGCAT |
| β-Actin | AGCACAGAGCCTCGCCTT | CATCATCCATGGTGAGCTGG |
